# Privacy and Security in Mobile Health (mHealth) Research

**DOI:** 10.35946/arcr.v36.1.15

**Published:** 2014

**Authors:** Shifali Arora, Jennifer Yttri, Wendy Nilsen

**Affiliations:** Shifali Arora, M.D., is an American Association for the Advancement of Science (AAAS) Fellow in the Directorate for Computer & Information Science & Engineering, National Science Foundation, Washington, DC. Jennifer Yttri, Ph.D., is an AAAS Science and Technology Policy Fellow in the Directorate for Computer & Information Science & Engineering, National Science Foundation, Washington, DC. Wendy Nilsen, Ph.D., is a Health Scientist Administrator in the Office of Behavioral and Social Sciences Research, National Institutes of Health, Bethesda, Maryland.

**Keywords:** Alcohol use, abuse, and dependence, problematic alcohol use, alcohol use disorders, mobile health, mHealth, wireless technology, mobile devices, sensors, data collection, intervention, privacy, security

## Abstract

Research on the use of mobile technologies for alcohol use problems is a developing field. Rapid technological advances in mobile health (or mHealth) research generate both opportunities and challenges, including how to create scalable systems capable of collecting unprecedented amounts of data and conducting interventions—some in real time—while at the same time protecting the privacy and safety of research participants. Although the research literature in this area is sparse, lessons can be borrowed from other communities, such as cybersecurity or Internet security, which offer many techniques to reduce the potential risk of data breaches or tampering in mHealth. More research into measures to minimize risk to privacy and security effectively in mHealth is needed. Even so, progress in mHealth research should not stop while the field waits for perfect solutions.

The recent proliferation of wireless and mobile health (mHealth) technologies presents the opportunity for scientists to collect information in the real-world via wearable sensors. When coupled with fixed sensors embedded in the environment, mHealth technologies produce continuous streams of data related to an individual’s biology, psychology (attitudes, cognitions, and emotions), behavior and daily environment. These data have the potential to yield new insights into the factors that lead to disease. They also could be analyzed and used in real time to prompt changes in behaviors or environmental exposures that can reduce health risks or optimize health outcomes. This new area of research has the potential to be a transformative force, because it is dynamic, being based on a continuous input and assessment process. Research in mHealth can ensure that important social, behavioral, and environmental data are used to understand the determinants of health and to improve health outcomes and prevent development of alcohol use disorders (AUDs).

Despite its promise, research in mHealth has progressed much more slowly than developments in industry. One reason is that issues of privacy and security remain an ongoing concern for researchers conducting mHealth studies, especially in areas involving sensitive behavior or treatment (e.g., alcohol use). Not only is the sensitivity of the data an issue for privacy and security, but also the amount that can be collected using mobile devices. Because most mobile devices (including phones and sensors) are carried by the person and collecting data throughout the day, researchers are now able to begin thinking about big data at the level of the individual (Estrin 2014). Fusion of streaming biological, physiological, social, behavioral, environmental, and locational data can now dwarf the traditional genetics and electronic health records-based datasets of so-called big data. Further, previously underserved groups can now participate in research because of the rapid adoption of mobile devices. In contrast with the Internet digital divide that limited the reach of computerized health behavior interventions for lower socioeconomic groups, mobile phone use has been rapidly and widely adopted among virtually all demographic groups (Pew Research Internet Project 2014). Now, 90 percent of American adults and 78 percent of teenagers have a cell phone, and more than half are smartphones (Pew Research Internet Project 2014).

Many of the strengths of mHealth research (i.e., its ability to reach large and broad samples and collect continuously streaming data on a range of potentially sensitive and possibly illegal behaviors and events) also drive privacy and security concerns. These topics, as well as confidentiality, are all separate yet connected issues that researchers must address in protecting research participants. The National Committee for Vital and Health Statistics describes the differences between and among privacy, confidentiality, and security this way:

*“Health information privacy is an individual’s right to control the acquisition, uses, or disclosures of his or her identifiable health data. Confidentiality, which is closely related, refers to the obligations of those who receive information to respect the privacy interests of those to whom the data relate. Security is altogether different. It refers to physical, technological, or administrative safeguards or tools used to protect identifiable health data from unwarranted access or disclosure* (Cohn 2006).*”*

These issues are further complicated by Federal regulations governing personal health information, as well as sensitive information concerning alcohol, drug use or mental health. There also are many legal and ethical concerns about mHealth, especially when used to study alcohol, drug use or mental health. Among these issues is safety of participants and liability of researchers if a study participant experiences an emergency during the study (Kramer et al. 2014). Legal and ethical considerations should be discussed further by the mHealth community but will not be reviewed here. Instead, this article focuses on privacy, confidentiality, and security in mHealth, areas ripe with research questions and opportunities whose times are overdue.

## Federal Regulations Affecting Health Information Privacy and Security

Any study related to alcohol use generally must abide by several layers of Federal rules instituted to protect patients and research subjects.

### HIPAA

Regulations have been in place for close to 20 years surrounding the privacy of personal health information. In 1996, the Department of Health and Human Services—specifically the Office for Civil Rights—introduced the Health Insurance Portability and Accountability Act (HIPAA). Although research activity is not directly addressed in HIPAA, many researchers are employed by or work within HIPAA-covered entities and work under the HIPAA guidelines for privacy and security, especially when personal health information is being used. Title II of HIPAA defined policies and guidelines for maintaining privacy and security of a patient’s health information (U.S. Department of Health and Human Services 1996). Within Title II lies the Privacy Rule, the first set of national standards for protecting every individual’s health information, as well as the Security Rule, which set a national standard for protecting personal health information in an electronic format (U.S. Department of Health and Human Services 1996). At the time these rules were introduced, clinical health information existed primarily in the form of handwritten patient health records. Information generally was shared between care providers over the phone, by fax or in person. Consequently, initial regulations and guidelines focused on the challenges surrounding protecting information in these limited-sharing formats.

The regulations have evolved over the last 15 years as the needs of the healthcare system have changed. As systems have begun to use electronic health records, the guidelines have been amended to take new factors into consideration. Significantly, some components have not been modified: the rules still require authorization from the individual to share his or her personal health information; and an individual has the right to ask for and receive his or her own health information. Other areas have evolved: the security regulations now include updated administrative, physical and technical safeguards for protected health information (U.S. Department of Health and Human Services 2009*a*). The latest 2013 update, which expanded HIPAA through the HITECH Act Subtitle D, now allows a patient to receive protected health information in any electronic format preferred. The onus of protection has been extended beyond the initial group of “covered entities” (i.e., medical care providers, hospitals and insurance companies) to include those involved with Electronic Health Record (EHR) development and records management (U.S. Department of Health and Human Services 2013).

### The Common Rule

In addition to HIPAA, researchers must abide by the Federal Policy for the Protection of Human Subjects, also known as the Common Rule. The Common Rule was introduced in 1991 to protect individuals participating in research activities (U.S. Department of Health and Human Services 2009*b*). The Common Rule sets out detailed policies and guidelines about informed consent, adverse events, handling of biological data, and vulnerable populations, among other issues. An updated version of the Common Rule is undergoing review (U.S. Department of Health and Human Services 2011). One proposed change of significance to mobile health researchers is the addition of specific guidance on data security and privacy. If enacted as proposed, data privacy and security protections that would be applied to research on human subjects would be calibrated to the level of identifiability of the information being collected. Because standards for digital privacy and security were not delineated in earlier versions of the Common Rule, Institutional Review Boards were often asked to make judgments about topics for which they may not have had the proper expertise. Thus, standardizing requirements will allow for more uniformity in research review and more clarity for researchers as they design research protocols to support digital privacy and security.

### 42 Code of Federal Regulations Part 2

The field of alcohol and substance use research is unique in that a set of specific Federal regulations guides it above and beyond the requirements of HIPAA and the Common Rule. Under 42 Code of Federal Regulations Part 2 (42 CFR), the confidentiality of the records of patients with alcohol and substance abuse/dependence is man dated (http://www.ecfr.gov/cgi-bin/text-idx?c=ecfr;sid=af45a7480ecfb95bc813ab4bbd37fb5b;rgn=div5;view=text;node=42%3A1.0.1.1.2;idno=42;cc=ecfr). Alcohol and drug abuse records can only be shared after written consent is obtained from patients, even if the use of such records by healthcare professionals occurs in a medical emergency. CFR42 also prohibits the disclosure of a research participant’s identity in any report or publication, even with consent. Because of the sensitive nature of the personal health information involved, protection of privacy, security and confidentiality warrants extra thought by alcohol researchers.

## Responsibility to Protect Privacy and Security

Regulations governing privacy and security—while layered and complex—tend to hold few surprises for experienced research teams. Patient expectations related to privacy on mobile devices, however, offer a new challenge that study protocols must address. For example, research has shown that a majority of Americans (78 percent) consider information stored on their mobile phones to be as or even more private than the information stored in their personal computers (Urban et al. 2012). Although people believe that information on their phones is under their control, this is not always true. The settings on phones may allow applications to access and share more information than people realize. Research participants, by contrast, are told the truth about phone privacy and security issues—primarily that there are potential dangers that often center on data breaches. This apparent disconnect between perception of privacy in daily life compared with research settings is important. It suggests that broad efforts at enhancing technological literacy are needed, or researchers risk making mHealth applications seem less safe than other protected mobile activities, such as banking. Instead of voicing concerns about highlighting the risks in health research and care, the scientific community should support overall efforts to increase the public’s knowledge of privacy and security risks regarding technology, thus allowing a rising tide of literacy to float all mobile device–using boats.

As is the case in all research, privacy, confidentiality, and security policies should be created in advance of a project by developing written standard operating procedures. Developing a priori practices and principles of conduct for mHealth research projects is a crucial step in enhancing data and participant safety. Since the majority of security breaches in healthcare (not just mHealth) are due to unauthorized access to a device or from mishandling or misusing data (Bennett et al. 2010), mHealth researchers need to conduct a risk assessment to identify potential vulnerabilities as they develop and implement their systems. When designing and implementing a security plan to protect participant information, researchers should tailor the plan to fit the risks associated with their protocol. A plan for privacy and security safeguards should balance the type of information being used, the intended use of the mHealth tool, the method of sharing information, and the costs of the protections to develop a feasible system with the minimal amount of privacy and security risk.

## Privacy in mHealth

In the United States, privacy is considered an essential freedom. It is the right of individuals to determine for themselves when, how, and to what extent personal information is communicated to others. Because privacy targets the human side of information protection, the solutions to these issues target the humans using the technology. At the highest level, patients currently regulate who can access their personal health information through consent. The consent gives participants appropriate knowledge of what data are being collected, how they are stored and used, what rights they have to the data, and what the potential risks of disclosure could be. Unfortunately, as noted earlier, technological literacy in the United States limits people’s understanding of the true risks and benefits of mobile technology.

Because changes in technological literacy take time to implement, researchers in mHealth will need to develop systems that enhance participant privacy. More specifically, this means building mHealth systems that allow research participants some control over the data, whether this be control over which data are collected or over which data are released to the research team. Researchers will need to be explicit about the data they are collecting and what control the participants will have over it. This also means that mHealth researchers should be thoughtful about what research data they will collect.

An example of offering such patient control comes from the field of computer science. Although not a standard for other scientific areas in health, in a participatory model of research proposed in computer science (Shilton 2012), participants pick and choose which data to share, whether before data collection or after data have been sampled. A simple electronic or paper checklist of possible data points administered before data collection and/or a patient-facing data dashboard will allow participants to exercise their rights to control and access their data. Thus, which data are shared and which are held becomes a personal decision. This does create potential havoc for the design of data collection and analytic plans, but it has the advantage of ensuring that participants are thoughtful about the specifics of their privacy. It has the added benefit of helping participants learn about the privacy options available in their non-research mobile world, which, again, should enhance technological literacy.

Another option is to create a context-aware system that the participant controls. In such a system used for eHealth research, the privacy options change based on factors such as location and who is accessing the data to match the participant’s level of trust (e.g., Ruotsalainen et al. 2014). Although limited, the work in patient-controlled data access has shown that most people who participate will not cull their data once they have committed to a study. The best practice may therefore yield greater satisfaction with the research process, because privacy is seen as protected in accordance with patient preference but results in minimal impact on data collection or the analytic plan.

mHealth also poses privacy challenges from people not enrolled in the research. Examples of this issue include the use of mobile cameras or microphones to collect data, but which also pick up sounds and images from non-participants. As with the issues raised at the participant level, ways to address these problems are needed. Solutions can be found not only at the level of study design but also through the use of techniques that can extract information from raw data and abstract such information, thereby protecting privacy.

Confidentiality in mHealth research shares many of the same factors as conventional research. A research team should be aware of the need to keep personal information private and to release information only in aggregate. Researchers should also collect only the minimum amount and detail of data needed for their research to reduce the risk of reidentification. For mHealth, an additional concern arises through the frequent use of third-party developers to build systems, including the databases for the project. These developers may continue in a project to ensure the system is updated and performing appropriately. As with all research team members, the developers—who may have little or no experience with human subjects—will need a carefully considered educational plan to understand the privacy and confidentiality of health information, especially when the data target the sensitive subject of alcohol use.

## Security in mHealth

Security refers to the safeguards, techniques, and tools used to protect against the inappropriate access or disclosure of information. Research suggests that legitimate users of a system often may be the likely cause of impaired security when they overlook rules, because they underestimate or fail to understand the costs of their actions (Besnard and Arief 2004). Thus, when it comes to securing data, researchers should try to prevent the most likely breaches, such as leaving mobile devices unsecured, sharing passwords or leaving them written on notes, accessing sensitive information in public areas using open-WiFi networks, or even losing a mobile device. While outsiders may intentionally attempt to access information or try to figure out someone’s identity or location from intercepting communications, such efforts will account for a minority of security threats. Many breaches are preventable through having a high-quality security plan that pays special attention to the most common and simplest reasons for data losses.

The overall goal of effective security protocols is to protect participant identity and secure data in such a way that if unauthorized individuals were to gain access, they would be unable to link the data with a particular person or with other data being sent. This is especially true because while no single source of data may be identifiable, the combination of multiple sources of data may make identifiable linkages possible. In mHealth, information is often transmitted at a high frequency and transferred over wireless networks, which can be more susceptible to monitoring and interception than broadband (Internet) networks, making security protocols the only barriers protecting data against a breach (Luxton and Kayl 2012).

### Simple Protections and Encryption

As noted earlier, when creating a security protocol, simple ways to increase data security should be considered first. For example, enabling WPA2 encryption on a wireless device enhances the security of information transmitted over wireless networks, but it must be enabled on the mobile device. In all cases in which consumer devices are used (e.g., a mobile phone or tablet), the use of a password (e.g., S0briety!), numerical pin (e.g., 16479), or pass-phrase (e.g., G0 2 the moon with me!) is highly encouraged. Support for these techniques should be offered to participants at the start of a study, because they often either do not know the techniques for developing an effective password (e.g., not using the word “password”) or their lack of technological literacy may make them think that risks are low and cause them to discard safety features once they control the devices. Finally, researchers can enable remote data wiping or locking protocols on phones or tablets used for mHealth. These systems, which come standard on many operating systems or can be added to devices, allow data to be wiped remotely and the device locked if it is thought to have been lost or stolen.

Researchers also should consider carefully which data need to be transmitted and where they will be stored. For instance, medication adherence reminders can be developed without reference to specific drug categories or even a mention of disease. The WelTel Kenya study by Lester and colleagues (2010) ingeniously used the phrase “Mambo?” in an SMS message to HIV-infected individuals, which is Kiswahili for “How are you?” These messages did not mention disease or anti-retroviral drugs, which would have identified people as HIV infected. Instead, the messages, even if received by someone else, could convey the study team’s question without potentially jeopardizing any participant’s privacy and security.

Minimizing the potential impact of data breaches can also be achieved by not storing data on a mobile device. For example, if a protocol includes the development of a personal health record with detailed health data, the research team might consider encrypting data (see below) and storing it in a secure server for aggregation. Participants could access the data through a wireless network, but data would not be left in the device after the application closed.

Simple precautions are an effective part of a security protocol, but securing data also has technical aspects, which for many studies are essential to protecting and maintaining integrity and security. Many of the more complex technical challenges surrounding securing data have been addressed by the cybersecurity community, which can offer guidance and potential solutions (Bennett et al. 2010; Sorber et al. 2012). Some of these security models are discussed below.

Encryption of data is a key component of security that allows for the protection and preservation of anonymity, but it must be done before the transfer of data. This process hides the content of a message while it is in transit, and the original message can only be seen through a process called decryption. A shared “key” is needed in the process of encrypting and decrypting and in healthcare settings. According to Federal HIPAA and HITECH Act regulations, this key must contain 128-bits (i.e., the length of the key) to offer sufficient security (Department of Health and Human Services 2013). National and international encryption standards have been generated for mobile technology, and researchers should use these when developing encryption and decryption algorithms. NIST (the National Institute of Standards and Technology) recommends using Suite-B (https://www.nsa.gov/ia/programs/suiteb_cryptography/), a set of algorithms that employs the cutting edge for exchanging decryption keys and digital signatures to authenticate data (Adibi et al. 2013).

Once data are encrypted and the challenge of anonymity has been addressed, the data collected can be transferred. For some mHealth, using a VPN (Virtual Provider Network) is a highly secure way for the appropriate people to connect to data to be transferred. VPNs have been used frequently by Internet and eHealth communities (Adibi et al. 2013). However, for mobile devices, using a VPN may be challenging because of streaming data or because the system slows data transmission and may reduce the speed of user-supplied devices, both of which may add to participant burden.

In addition to VPNs, various mechanisms can be applied to protect data in transit. For example, data can be transferred in different orientations for further protection. Because this is an area of interest in the cybersecurity research community, multiple mechanisms to accomplish it have been created. The goal during transfer is to send the messages efficiently so they do not overwhelm the system, tag messages so they can be recognized only by the receiver, and make sure that no data tampering occurs (Mare et al. 2011*a*,*b*).

One important aspect to remember during security protocol development is that the higher the level of security, the greater the cost of the transmission in terms of time and encryption, as well as burden of use. Another method of securing the data during transfer is to change the strength of security depending both on the safety of the environment in which the data are being collected (i.e., a home versus a public area) and on the device the data are being sent from (trusted or not trusted) (Prasad and Alam 2006). Thus, a study might use a multiple-level strategy for EHR data being viewed on a mobile device, but not on single transmissions coming from devices using a secure network in the home. Location of data transfer and level of device trust would form part of a plan to help determine which level of security should be used and when.

### Authentication

Authentication ensures that the data collected are associated with the correct participant; that only authorized individuals have access to data and tools; that only valid and protected devices are used; and that data are sent through authorized channels. The cybersecurity community uses two-factor authentication as its current and usually consists of a PIN number, The last category, which only appears highest standard. In cybersecurity, password or passphrase. This is cur-in rare circumstances, is unique to there are three different categories for rently the most common mode of each user and includes fingerprints, authentication: “something known,” authentication. The second category eye scans or voice recognition. For “something possessed,” and “some-for authentication includes a tangible two-factor authentication to take thing unique to the person” (Varchol item that users can carry with them place, correct responses are required et al. 2008). The first is set by the user such as a token, smart card, or dongle. in two out of the three categories (Varchol et al. 2008). Two-factor authorization may not be needed in most research, but it should be considered when sensitive data with high potential negative impact are being transmitted. Many available authentication systems can be added to new mHealth tools (Adibi et al. 2013). Again, the first approach is to avoid or minimize the amount of high-impact data being transmitted.

### Risk Assessments and Audits of the Security System

Security breaches can occur at any stage of implementation of mHealth technology. As part of a research protocol, risk assessments should be included to ensure that the lowest possible risk to security is maintained. Audits of a security system are required as part of HIPAA, HITECH, and international security standards and should be performed throughout testing and use to ensure security measures are working. Audits can be a natural byproduct of security measures and help to identify potential risks in a system. For instance, authentication protocols for individuals and devices connecting to a system and accessing information leave an audit trail that automatically notes which participant’s personal health information was handled and by whom. This ensures that any failures in the system are detected and holds each insider accountable for following proper protocols to maintain privacy and security.

It is clear that when researchers combine multiple layers of safeguards to ensure privacy and security, they are better placed to protect personal health information. To determine whether such a layered system still contains security gaps, the best approach is to test it. A potential method for testing security that is used successfully in the cybersecurity world involves employing “red teams”, experts charged with hacking into cyber systems to assess weaknesses. Red teams can identify safety flaws before a technology is deployed, thereby preventing safety lapses. Although setting up official teams would add expense and burden to projects, researchers might be able to mimic this methodology by having non-involved research team members or graduate students in related programs (e.g., computer engineering and sciences) field test the technology or application before it is deployed to determine how easily the program can be disrupted or hacked. These efforts should be documented and communicated to the team and Institutional Review Board (IRB), as well as in grant applications and publications (as applicable). An example of successful risk assessment without the use of “red teams” comes from Henriksen and colleagues (2013). In designing their home-based eHealth platform, the project team used a brainstorming process to identify potential risks throughout the design and implementation process. They then applied simple measures to reduce those risks when deemed unacceptable at given stages in development.

## Moving Privacy, Safety and Security Forward in mHealth

Although security and privacy are critical, no system involving humans will be completely secure. Breaches will happen. Thus, a balance must be struck between security, subject usability and research cost based on the requirements of the mHealth research. The goal should be to mitigate security risks without impeding use and to set up a system that recovers from potential breaches. Safety protocols are available from other related fields that could be applied to mHealth. Protocols developed as standards for medical devices (Underwriters Laboratory ISO 14971; Underwriters Laboratory 2011) and ideas from the fields of telehealth, eHealth, and cybersecurity can be co-opted for use with mHealth products. For example, the Food and Drug Administration (FDA) has developed guidance for device safety and standards, publishing guidance documents designed to help developers generate safe and effective mHealth technology (FDA 2013). In practice, no common database of breaches of security for mHealth research exists, so actual patterns or typologies of these lapses, if they have occurred, have not emerged. If researchers experience a security lapse, there is no mechanism to report this beyond the university or even the research team. Thus, while secure systems are built to collect and manage mHealth data, what contributes to their success or failure remains unknown.

Further, because mHealth may have both novel risks and novel benefits, there is value to including community members—the people who will be most affected by mHealth technology—in discussions of privacy, safety, and security. Improving awareness and offering training in technological literacy, as noted earlier, are ways to reduce privacy and security risks caused by participants and increase involvement in mHealth. Many security features require input from the end user, and therefore education can help ensure the security of mHealth. Security training can be included with training for using mHealth tools and with education on the benefits of mHealth.

More research into measures to effectively minimize risk to privacy and security in mHealth is needed. While lessons can be borrowed from other communities, such as cybersecurity or eHealth, the unique challenges associated with mobile technology warrant development of novel security approaches. In the meantime, we have the knowledge to prevent privacy and security breaches while maintaining the benefits of using mHealth (see table). Progress in mHealth research should not stop while waiting for perfect solutions.

## Figures and Tables

**Table:** Addressing confidentiality, privacy, and security challenges in mHealth. Many risks may occur in design and use of mHealth. Solutions that are cost-effective and can be implemented without interfering with research are recommended to mitigate these risks. These solutions are commonly used in Internet/eHealth, telemedicine, and cybersecurity research.

Risk	Solution
**De-identification**	Share data in aggregate Separate transmission of identifying information (name, location) from other data
**Consent**	Use consent to educate participants about what data are being collected and what can be inferred from such data Include privacy and safety training for participants Consider allowing patients to choose which data to share and with whom
**Breaches from intended user**	Enable password, pin, or passphrase on phones before distribution Enable remote wiping
**Encryption**	Use WPA2 and 128-bit key encryption Add a tag or header to the encrypted message
**Data transmission**	Use non-sensitive messages to contact participants Store data remotely, such as on a secure server or in a cloud
**Data accessibility**	Store critical data in two locations to ensure availability
**Data integrity and quality**	Have a second system to collect the same data, such as in-person visits or surveys, to verify mobile data integrity and quality
**Location**	Have adjustable security settings for trusted and untrusted locations
**Authentication**	Use two-factor authentication, such as with a pin/password and a token/smart card/dongle
**Audits and risk assessment**	Include audits in security protocols, potentially with the help of a “red team”; risk assessment should be done at each stage of implementation
